# Animal alphacoronaviruses found in human patients with acute respiratory illness in different countries.

**DOI:** 10.1080/22221751.2022.2040341

**Published:** 2022-02-27

**Authors:** Anastasia N. Vlasova, Teck-Hock Toh, Jeffrey Soon-Yit Lee, Yong Poovorawan, Phillip Davis, Marli S. P. Azevedo, John A. Lednicky, Linda J. Saif, Gregory C. Gray

**Affiliations:** a Center for Food Animal Health, Department of Animal Sciences, College of Food, Agricultural and Environmental Sciences, The Ohio State University, Wooster, USA; b Clinical Research Center, Sibu Hospital, Ministry of Health Malaysia, Sibu, Malaysia; c Faculty of Medicine, SEGi University, Kota Damansara, Selangor, Malaysia; d Center of Excellence in Viral Hepatitis Research, Department of Pediatrics Faculty of Medicine, Chulalongkorn University, Bangkok, Thailand; e MRIGlobal, Kansas City, USA; f National Center for Toxicological Research, Division of Microbiology, U.S. Food and Drug Administration, Jefferson, USA; g Emerging Pathogens Institute, University of Florida, Gainesville, USA; h Department of Environmental and Global Health, College of Public Health and Health Professions, University of Florida, Gainesville, USA; i Departments of Internal Medicine (Infectious Diseases), Microbiology and Immunology, and Preventive Medicine & Population Health, University of Texas Medical Branch, Galveston, USA

**Keywords:** Canine coronaviruses, novel human alphacoronavirus, pneumonia, acute respiratory illness, zoonotic disease, East Malaysia, Haiti, USA

## Abstract

Here we review the existing evidence of animal alphacoronaviruses (*Alphacoronavirus 1 species*) circulating in human patients with acute respiratory illness. Thus far, the viruses similar to canine, feline and porcine alphacoronaviruses (including the most recent CCoV-HuPn-2018 and HuCCoV_Z19) have been detected in humans in Haiti, Malaysia, Thailand, and USA. The available data suggest that these viruses emerged in different geographic locations independently and have circulated in humans for at least 20 years. Additional studies are needed to investigate their prevalence and disease impact.

## Introduction

The recent emergence, rapid spread, and tremendous public health impact of SARS-CoV-1, MERS-CoV, and SARS-CoV-2 underscore the premise that animal coronaviruses (CoVs) are a much more serious public health threat than we estimated 20 years ago [1, 2]. A better understanding of their prevalence, interspecies transmission mechanisms and pathogenesis is urgently needed.

Recently, we isolated and determined the complete genome sequence of a novel canine-like alphacoronavirus (CCoV-HuPn-2018) from a hospitalized infant with pneumonia in Sarawak, Malaysia [3]. Complete genome sequence analysis demonstrated that the CCoV-HuPn-2018 genome had a typical recombinant (canine-feline-porcine) structure of many previously characterized canine coronaviruses (CCoVs) of genotype II but possessed some unique genomic features (such as 12aa deletion in its N protein). These data provided the first substantial evidence that canine-feline-porcine-like (CFPL) CoVs of *Alphacoronavirus 1* species can infect and be associated with acute respiratory illness in humans (hCFPL-CoVs).

Besides our recent report, we are aware of three additional studies that identified similar hCFPL-CoVs in patients with acute respiratory illness [4–6]. Those include: a) a study from Thailand that detected hCFPL-CoVs among eight pediatric patients (outpatient or hospitalized) with acute lower respiratory tract infections (ALRTI) in Bangkok (Thailand) in 2002–2003 [5], b) a study from the USA (Arkansas) that identified hCFPL-CoVs in three influenza negative patients with acute flu-like illness in 2010 [4], and c) a study that identified hCFPL-CoVs in six patients who visited Haiti with a missionary trip in 2017 and developed mild fever and malaise upon returning to the USA [6]. While these findings do not necessarily prove that hCFPL-CoVs are pathogenic to humans, we emphasize that each hCFPL-CoVs detection was reported in association with acute respiratory illness. In contrast, no reports on hCFPL-CoV circulation in healthy humans are currently available. Additionally, while two studies (USA and Malaysia) have confirmed the presence of additional respiratory pathogens, hFCPL-CoVs were the only pathogens identified in the other two studies (Haiti and Thailand) [3–6].

The above surveillance studies (in Malaysia, USA and Thailand) demonstrated the prevalence of hCFPL-CoVs varying between 1.5 and 3.54% among patients with acute respiratory illness [3–5,7]. While hCFPL-CoV prevalence among healthy humans is not known, it is concerning, that many of the hCFPL-infected patients presented with pneumonia [3,5].

Of interest, the earliest identification of hCFPL-CoVs in pediatric patients with ALRTI in Thailand resulted in their misclassification as human CoV 229E (HCoV 229E), while the study found that it was the prevalent CoV infection associated with ALRTI in children [5]. During our recent molecular analysis of CCoV-HuPn-2018, these “human CoV 229E” variants were found to be closely related to CCoV-HuPn-2018 (and other CCoVs), but phylogenetically distant from other HCoV 229E strains. The original misclassification has likely occurred due to the scarcity of alphacoronavirus complete genome sequences at the time of the study performance and its manuscript publication.

In 2010, Silva et al. identified hCFPL-CoVs in one pediatric (<1–10yrs) and two senior (>61yrs) (out of 200) patients with acute influenza-like illness in Arkansas, USA [4]. Partial ORF1ab and S sequences were obtained, and sequence analysis demonstrated that they shared the highest NI with different feline CoV (FCoV) strains: ≥93.49% (Hu-131, ORF1ab), ≥86.5% (Hu-139, ORF1ab), ≥93.62% (Hu-142, ORF1ab) and ≥87.46% (Hu-142, S).

Most recently, Lednicky and colleagues isolated a novel CCoV (HuCCoV_Z19) from a medical team member who presented with fever and malaise upon returning from Haiti and demonstrated that it shared 99.4% nt identity with CCoV-HuPn-2018 at the complete genome level [6]. Thus, while our recent study demonstrated that hCFPL-CoV-infected patients were mostly young children (7 out 8 patients) [3], Lednicky and colleagues provided further evidence that hCFPL-CoVs can be associated with mild illness among adults [6]. HuCCoV_Z19 genome had a recombinant structure similar to CCoV-HuPn-2018 and evidence of further recombination events that could have occurred in human hosts. Besides CCoV-HuPn-2018, HuCCoV_Z19 is the only other hCFPL-CoV that was isolated in cell culture and which complete genome sequence was determined, as the studies from USA and Thailand only generated partial sequences from the polymerase gene complex.

Here we conducted further analyses to understand the genetic relationship between the known hCFPL-CoVs and their genetic heterogeneity.

## Results and discussion

**Figure 1:**
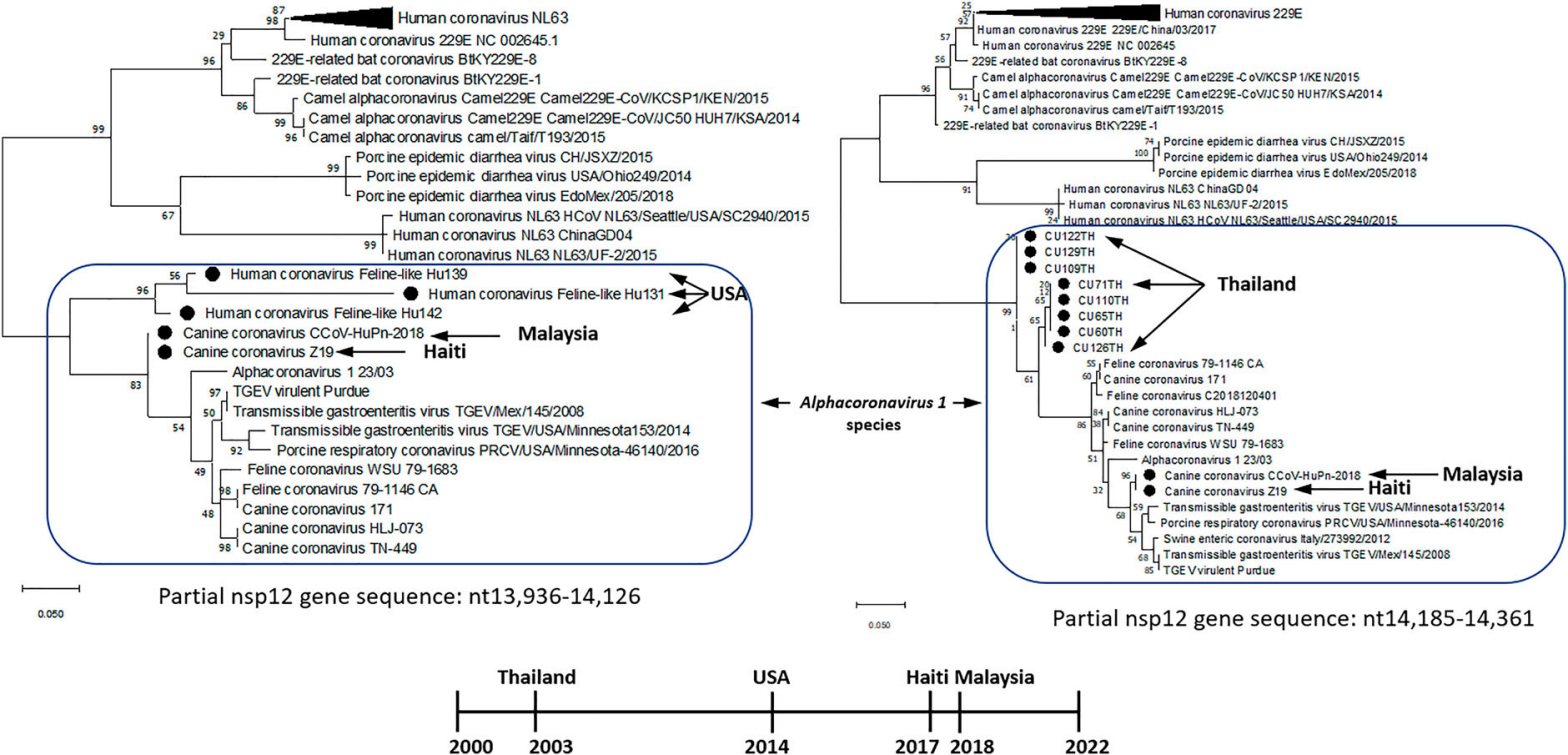
. Phylogenetic tree of hCFPL-CoVs based on the nsp12 region available for CU65TH-CU129TH samples from Thailand **(A)**, and on the nsp12 region available for Hu139-Hu142 samples from USA **(B)**. Bootstrap values are represented at key nodes. Scale bar indicates nucleotide substitutions per site. The evolutionary history was conducted in MEGA X inferred using the Maximum Likelihood method and General Time Reversible model. The hCFPL-CoVs are labeled with black circle marker.

Phylogenetic and sequence comparison analyses demonstrated that all identified hCFPL-CoVs represent a group of heterogeneous but closely related CoVs originating from CCoVs, FCoVs and transmissible gastroenteritis virus (TGEV) of *Alphacoronavirus 1* species **(**Figure 1, Table 1 near here**)**. All hCFPL-CoVs from different geographic locations formed a monophyletic branch within *Alphacoronavirus 1* species, while all other alphacoronaviruses of human or animal origin formed separate clusters (Figure 1). Unfortunately, partial ORF1b sequences of hCFPL-CoVs from Thailand and USA were derived from non-overlapping regions of the nsp12 gene, 13,936–14,126 (191 bp) and 14,185–14,361 (176 bp), respectively, so no direct comparison between these two sets of sequences could be performed.

**Table 1:** . Nucleotide identity among hCFPL-CoVs and other animal and human alphacoronaviruses.

	CCoV-HuPn-2018 (Malaysia)	HuCCoV-Z19 (Haiti)	CU65TH-CU129TH (Thailand)	Human feline-like CoV strains Hu131, Hu139 and Hu142 (USA)	CCoV*	FCoV*	TGEV*	HCoV 229E
Complete genome								
CCoV-HuPn-2018 (Malaysia)	100%	99.4%	N/A	N/A	93.74%	84.58%	92.29%	53.67%
HuCCoV-Z19 (Haiti)	99.4%	100%	N/A	N/A	93.86%	83.1%	92.4%	53.47%
ORF1ab region available for CU65TH-CU129TH (Thailand)								
CCoV-HuPn-2018 (Malaysia)	100%	100%	87.93–89.66%	N/A	≤94.88%	≤93.36%	≤93.95%	≤73.95%
HuCCoV-Z19 (Haiti)	100%	100%	87.93–89.66%	N/A	≤94.88%	≤93.36%	≤93.95%	≤73.95%
CU65TH-CU129TH (Thailand)	87.93–89.66%	87.93–89.66%	97.13–100%	N/A	≤95.91	≤95.91	≤91.81	≤77.59%
ORF1ab region available for Human feline-like CoV strains Hu131, Hu139 and Hu142 (USA)								
CCoV-HuPn-2018 (Malaysia)	100%	100%	N/A	≤86.98%	≤99.43%	≤95.98%	≤98.26%	≤73.56%
HuCCoV-Z19 (Haiti)	100%	100%	N/A	≤86.98%	≤97.13%	≤95.98	≤98.26%	≤73.56%
Human feline-like CoV strains Hu131, Hu139 and Hu142 (USA)	≤86.98%	≤86.98%	N/A	81.4–97.9%	≤86.98%	≤93.49%	≤84.19%	≤73.02%

*Highest nucleotide identity shown was shared between hCFPL-CoVs and variable CCoV, FCoV and TGEV strains.

Sequence comparison analysis of the partial genomic sequences of hCFPL-CoV strains from Thailand and the USA demonstrated that they shared only 86.9889.66% NI with the same genomic regions of CCoV-HuPn-2018 and HuCCoV_Z19 (Table 1), while CCoV-HuPn-2018 and HuCCoV_Z19 were 100% and 99.4% identical within those regions and at the complete genome level, respectively. Lower NI among hCFPL-CoVs (except for highly similar CCoV-HuPn-2018 and HuCCoV_Z19) coinciding with higher NI between the individual hCFPL-CoVs and different strains of CCoV, FCoV and TGEV (Table 1) suggestive of multiple spillover events of the latter animal CoVs into human populations in different geographic regions or time frames. Of interest, most CCoV and FCoV strains closely related to hCFPL-CoVs were of genotype/serotype II. Additionally, comparison of the two different genomic fragments (available for Thailand and USA strains) of CCoV-HuPn-2018 and HuCCoV_Z19 demonstrated they had variable levels of NI with CCoV, FCoV and TGEV strains (Table 1) consistent with the history of multiple recombinant events among these strains. However, NI with HCoV 229E (Table 1) and other human alphacoronaviruses (not shown) remained consistently low suggestive of independent evolution of hCFPL-CoVs.

In summary, prior and current data suggest that genetically heterogenous hCFPL-CoVs are associated with respiratory illness in humans circulate on different continents. Because hCFPL-CoVs from different countries and years share higher NI with various CCoVs, FCoVs and TGEVs, no single ancestral strain can be implicated in the global emergence of the different hCFPL-CoVs. High genetic heterogeneity within and between different groups of hCFPL-CoVs identified suggests that cluster infections with hCFPL-CoVs may occur as individual zoonotic spillover events. *However, the high genetic similarity between CCoV-HuPn-2018 and HuCCoV_Z19 (from geographically distant Malaysia and Haiti but closely timed) may be indicative of stronger temporal and weaker spatial influences driving hCFPL-CoV evolution in the human host.* Further studies of hCFPL-CoV prevalence, human-to-human transmission, pathogenic potential, and genetic composition are a high priority considering the current evidence of hCFPL-CoV association with potentially severe respiratory illness in humans. Thus, this analysis once again emphasizes the importance of continual surveillance to identify zoonotic CoVs (as well as other viruses) at the human-animal interface [8–10].

## Materials and methods

To further clarify the relationships among the identified hCFPL-CoVs, members of *Alphacoronavirus 1* species, and other human and animal alphacoronaviruses, we have conducted phylogenetic and sequence comparison analyses for the available genomic parts (complete genomes for CCoV-HuPn-2018 and HuCCoV_Z19 and partial ORF1ab sequences for variants from the USA and Thailand). The analysis included the following CoV sequences: FCoV strains (MW030109.1, JN634064.1, MN528741.1); CCoV strains (KC175339.1, KY063618.2, JQ404410.1); CCoV-HuPn-2018 (MW591993.2); HuCCoV_Z19 (MZ420153); TGEV strains (DQ811789.2, KX900410.1, KX900402.1); swine enteric CoV strain Italy/273992/2012 (KT027396.1); HCoV 229E (MT797634, JX503060.1, KY996417.1, NC_002645.1, KY674919, MW532107, MT438699.1, MN306046.1, MF542265.1, MT438700.1, MW532103.1, KU291448.1); HCoV NL63 strains (MK334047.1, KX179500.1, KY983586.1); porcine epidemic diarrhea virus strains (MT490316.1, MT625963.1, KR265840.1), camel alphacoronavirus Camel 229E strains (KT253328.1, KU291449.1, KT368916.1); porcine respiratory CoV (KY406735.1); Alphacoronavirus 1 strain (KP849472.1); 229E-related bat CoV strains (KY073748.1, KY073747.1); HCoV feline-like strains Hu131, Hu139 and Hu142 (KF524841, KF524840, KF524843.1, KF524853); SARS-CoV-2 (NC_045512.2); misclassified HCoV 229E strains from Thailand [CU60TH (AY751815), CU65TH (AY751814), CU71TH (AY754762), CU109TH (AY751812), CU110TH (AY751816), CU122TH (AY751811), CU126TH (AY751813) and CU129TH (AY751810)].

Sequence comparison analysis was conducted using BLASTn and Sequence Manipulation Suite (SMS, Version 2, https://www.bioinformatics.org/sms2/).

### Statements

* This manuscript reflects the views of the authors and does not necessarily reflect those of the Food and Drug Administration.
